# Real-world short-term outcomes after switching to fixed-interval anti-VEGF therapy for neovascular AMD during the COVID-19 pandemic

**DOI:** 10.1186/s40942-025-00734-w

**Published:** 2025-10-08

**Authors:** Arnulfo Garza Reyes, Tirth J. Shah, Edward F. Linton, Zachary Q. Mortensen, Timothy M. Boyce, Aaron M. Ricca, Razek G. Coussa, H. Culver Boldt, James C. Folk, Stephen R. Russell, Ian C. Han, Karen M. Gehrs, Elliott H. Sohn

**Affiliations:** 1 Department of Ophthalmology and Visual Sciences, University of Iowa Health Care, 200 Hawkins Dr, Iowa City, IA 52242 USA; 2 VA Center for the Prevention and Treatment of Visual Loss, Iowa City, IA USA

**Keywords:** Age-related macular degeneration (AMD), COVID, Treat-and-extend, Fixed-interval, OCT, Neovascular, Macular neovascularization, CNV, Wet AMD

## Abstract

**Purpose:**

To assess short-term real-world visual and anatomic outcomes in patients with neovascular age-related macular degeneration (nvAMD) following a protocol shift from an individualized treat-and-extend (T&E) anti-VEGF regimen to a fixed-interval injection schedule without routine imaging during the early COVID-19 pandemic.

**Methods:**

This retrospective cohort study included patients with nvAMD undergoing anti-VEGF therapy at a single tertiary academic retina practice. In response to the COVID-19 pandemic, the clinic transitioned from a T&E approach to a fixed 8-week injection schedule without routine OCT imaging between March 25 and July 1, 2020. Eyes with at least two visits and OCT imaging before and after the protocol change were included. Outcomes measured included visual acuity (VA), central macular thickness (CMT), fluid status, appointment adherence, COVID-19 cases, and mortality. Statistical comparisons were performed using paired t-tests and Wilcoxon signed-rank tests.

**Results:**

A total of 289 eyes (232 patients) were included. Mean VA before the switch was 20/45 (logMAR 0.35 ± 0.33) and 20/50 (logMAR 0.39 ± 0.33, *p* = 0.17) after the switch. Mean CMT showed non-significant changes from 292.1 ± 89 μm pre-switch to 298.7 ± 66 μm (*p* = 0.15) post-switch. COVID-19 affected 11 patients (6 deaths); no cases were linked to clinic visits, and staff remained unaffected.

**Conclusions:**

Switching to fixed-interval injections with limited imaging effectively maintained visual and anatomical outcomes while safely reducing COVID-19 transmission risks. These results suggest that fixed-interval regimens may preserve short-term outcomes in established nvAMD patients and could be useful during future care disruptions or protocol changes.

**Supplementary Information:**

The online version contains supplementary material available at 10.1186/s40942-025-00734-w.

## Introduction

Age-related macular degeneration (AMD) is the leading cause of severe, irreversible vision loss among patients older than 50 years [1]. Before the advent of anti-vascular endothelial growth factor (anti-VEGF) intravitreal injections [2–4], the neovascular form of AMD (nvAMD) was responsible for more than 80% of cases of severe visual loss or legal blindness [5]. The visual changes associated with nvAMD are relatively rapid and can be irreversible if it is not treated early, making periodic examinations and treatment with intravitreal injections of paramount importance.

The SARS-CoV-2 virus (COVID-19) pandemic significantly altered the options available to healthcare providers offering outpatient care in the spring of 2020 in the United States [6]. In an attempt to comply with regulatory guidance to reduce viral transmission, there were unprecedented cancellations of outpatient clinic appointments and a shift to telemedicine was seen [7 Our hypothesis was that patients with nvAMD dependent on scheduled anti-VEGF injections were particularly vulnerable to potentially severe, irreversible vision loss during this time period.

To address the treatment challenges, a variety of algorithms and guidelines were implemented in different countries, states, counties and hospitals. Many practices worldwide were forced to close their clinics or deny care entirely, resulting in substantial treatment delays, sometimes lasting many months [7, 8]. Some tertiary centers adopted a priority system that determined intravitreal injection intervals based on initial disease activity and visual acuity loss [9]. Other practices adopted a treat-and-extend (T&E) protocol where sequential injections were extended by 2 weeks while some adhered to a treat-as-needed (PRN) protocol based on telephone triage [10]. Despite some published literature on the heterogeneous protocols used during the pandemic, there remains limited data on outcomes associated with these various protocols; thus, specific experiences at institutions with clearly-defined protocols provide valuable information regarding the potential impact on such changes in the case of potential future pandemics.

Our department’s approach attempted to minimize patient risk from viral infection and maximize maintenance of vision. On March 25th 2020, the Retina Service at the University of Iowa altered its injection protocol from treat-and-extend based on history, retinal exam, and OCT imaging to a fixed 8 week interval injection schedule with highly limited OCT imaging. During the study interval, our service also shifted all patients requiring intravitreal injections for nAMD from a multiple location/multiple provider format to a single clinic location with the sole function of providing intravitreal injections by just two providers (faculty and fellow). Few studies have specifically examined the effects of a deliberate, clinic-wide shift from T&E to fixed-interval dosing without routine imaging in an established nvAMD population. In this study, we evaluated short-term visual and anatomic outcomes following a real-world protocol change from T&E to fixed-interval anti-VEGF therapy at a single tertiary retina center during the early months of the COVID-19 pandemic.

## Methods

This study protocol was approved by the Institutional Review Board (#202106016) of University of Iowa and was performed in accordance with the tenets of the Declaration of Helsinki.

### Study participants

This retrospective study included a chart review of all patients with nvAMD at the University of Iowa Health Care, Department of Ophthalmology and Visual Sciences. Standard care treatment at our institution for nvAMD includes a treat-and-extend regimen based on patient history, social determinants of health, visual acuity, and evidence of disease activity including on exam and OCT imaging. During the height of the COVID-19 pandemic, we instituted a department-wide fixed 8 week interval injection schedule for our patients from March 25th, 2020 to July 1st, 2020 (termed ‘protocol switch’). A reduction of testing was implemented because optical coherence tomography (OCT) and fundus photos, which places the photographer and patient in relatively close proximity, could increase the likelihood of exposure for virus transmission and cause contamination of the instruments. Therefore, OCTs were performed only on an as-needed basis when there were (1) new subjective vision symptoms, (2) a drop in visual acuity (VA) of more than two Snellen lines or (3) concerning findings on indirect ophthalmic exam such as new hemorrhage. This was a departure from our pre-protocol switch injection clinic workflow where all patients had OCTs performed before evaluation by the treating physician. In addition, patients had their intake with history, visual acuity check, ophthalmic exam, and injection procedure performed in the same clinic room chair to limit potential virus transmission.

The inclusion criteria for patients in this study included evidence of nvAMD diagnosed by a board certified, retina-fellowship trained attending physician and history of recurring anti-VEGF therapy. An Epic electronic health records query was performed for patients with the following International Classification of Disease (ICD) – 10 codes: H35.3211, H35.3212, H35.3212, H35.3221, H35.3222, H35.3223, H35.3231, H35.3232, and H35.3233. Patients were required to have at least one visit prior to the pandemic protocol switch (3/25/20) and one visit after resuming regular operations (7/1/20). Exclusion criteria for eyes included in this study were any maculopathy secondary to causes other than AMD or any documentation of vision loss not attributed to AMD, which included progression of cataract, progression of posterior capsular opacification, progression of ocular surface disease, retinal vascular occlusions, and/or other etiologies that could account for vision loss such as glaucoma and visually significant cataracts. Those with vision loss attributed to diabetic retinopathy were excluded. Patients who had new onset nvAMD after 3/25/20 were not included in this study. Lastly, patients with foveal-involving geographic atrophy or central atrophy from other causes were excluded from this study.

The study analyzed clinical data acquired from a patients’ last visit prior to 3/25/20, and first visit after 7/1/20. We documented the patient’s best-corrected VA using pinhole, central macular thickness (CMT), type of intravitreal medicine given, injection schedule, number of injections received from 3/25/20 to 7/1/20, and OCT evidence of exudative activity, as further outlined below. A three-month follow-up window was chosen to assess the immediate impact of the protocol change, reflecting the real-time clinical decisions made during the initial phase of the COVID-19 pandemic. VA measurements were made using the Snellen chart and converted to the logarithm of the minimum angle of resolution (logMAR) as described by Holladay and colleagues [11].

### OCT imaging and grading

Optical coherence tomography (OCT) imaging was performed prior to and during the pandemic switch (the last before March 25,2020) using the Heidelberg Spectralis HRA + OCT device (Heidelberg Engineering, Heidelberg, Germany) centered on the fovea with a 19 line scan volume of 20 × 20° cube at 1064 × 49 × 1024 voxels. OCT images after the protocol switch (first scan after July 1, 2020) were performed using the Cirrus (Carl Zeiss Meditec, Jena, Germany) macular cube centered on the fovea with 512 A-scans × 128 horizontal B-scans covering an area of 6 × 6 mm centered on the point of fixation. The change in OCT machine immediately after the protocol switch was due to technical staff preferences after a change in location for injections. These OCT images were graded by two masked, independent, experienced readers for qualitative features consistent with active exudatation, as previously described, including the presence of intraretinal fluid (IRF), subretinal fluid (SRF), and subretinal hyper-reflective material (SHRM) [12–14]. Structural OCT images were reviewed retrospectively using the softwares on the respective machines. Qualitative analysis of retinal imaging was compared with the documented interpretation of the OCT imaging in the patient chart for consistency. Any disagreement between the graders was resolved via an open discussion between the two graders and an independent vitreoretinal specialist.

The central 1 mm macular thickness (CMT) using either the Heidelberg Eye Explorer or Cirrus software depending on the device utilized were also recorded for each eye for the two visits of each patient in the study. The calculation of the CMT in both of this devices differs with the Spectralis measuring CMT from internal limiting membrane (ILM) to Bruch’s membrane, while the Cirrus measures it from ILM to the middle of the retinal pigment epithelium (RPE) [15–17]. Cirrus-derived CMT values were converted to Spectralis equivalents using a previously published conversion formula by Sun et al.: Spectralis CMT = 40.78 + (Cirrus CMT x 0.95) [18]. In eyes with pigment epithelial detachments (PEDs), this conversion may underestimate CMT compared with true ILM–Bruch’s membrane measurements. Manual re-segmentation to align the Cirrus lower boundary with Bruch’s membrane was not feasible for the entire cohort but could be performed in future validation studies. Scans with gross segmentation errors were excluded from analysis.

### Statistical analysis

The main outcome was change in VA at the first follow up visit after the protocol switch compared to baseline. For analysis, recorded Snellen VA was converted to LogMAR, and VA and CMT were compared between baseline and follow-up using mixed effects linear regression with fixed effects of LogMAR, VA, and CMT respectively, as well as a random effect of subject, with a compound symmetry covariance structure. This method was chosen to account for the correlation between eyes as both eyes of many subjects were included. The change in the presence of IRF, SRF, and SHRM before and after pandemic restrictions were assessed using McNemar test for paired nominal data [19]. The incidence of substantial vision loss (LogMAR increase of 0.3, corresponding to ~ 3 lines of vision loss) per eye-month was calculated and compared to an expected value derived from the literature using a chi-square test. A p-value of less than 0.05 was considered statistically significant. Statistical analysis was performed using Microsoft Excel (Microsoft, Redmond, WA) and Rstudio (Rstudio PBC, Boston, MA).

## Results

### Characteristics of patients included in the analysis

Two hundred eighty-nine (289) eyes from a total of 198 patients met the inclusion criteria for this study. Eyes were excluded if they had fovea-involving geographic atrophy (*n* = 42 eyes), if they were not actively receiving regularly scheduled injections (*n* = 51), had maculopathy secondary to causes other than AMD (*n* = 15), had progression of glaucoma (*n* = 9), significant progression of cataract (*n* = 7), or progression of ocular surface disease (*n* = 4). The average age of our cohort was 80.3 years. Approximately 39% of the patients in our analysis were males and 61% were females (Supplementary Table 1). Of all eyes, 120 received bevacizumab, 119 received aflibercept, 48 received ranibizumab, and 2 received brolucizumab. The average number of injections per patient was 1.89 +/- 0.30 during the 13-week protocol switch interval. By comparison, prior to the protocol switch, the average number of injections per patient was 2.12 +/– 0.40 per 13 weeks.

### Visual acuity analysis

**Fig. 1:**
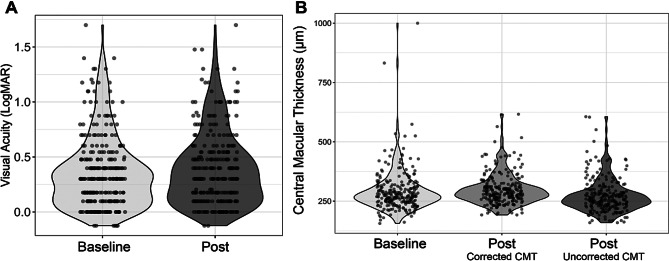
Comparison of visual acuity (VA; **A**) and central macular thickness (CMT; **B**) at baseline and at follow up (‘Post’) after the protocol switch to fixed-interval injections. Violin plots of (**A**) Best-corrected VA (logMAR) from our cohort did not significantly change over the three-month period of the protocol switch (increase in logMAR of 0.026 ± 0.019, *p* = 0.17) and (**B**) CMT which did not change with conversion of CMT to corrected values as OCTs done after the switch were performed on the Cirrus compared to Heidelberg at baseline

Mean VA before the pandemic switch was 20/45 with 5.2% worse than 20/200. Mean VA after the switch was similar: 20/50; with 4.5% worse than 20/200 (more details in Fig. 1A). Mixed effects linear regression comparing the effect of timepoint (follow-up vs. baseline) on VA showed no significant difference in VA before and after the protocol switch. (β = 0.026 ± 0.019 logMAR units, corresponding to a few letters of worsening; *p* = 0.17). Overall, patients maintained vision throughout the three-month period of the protocol switch.

### OCT analysis

The mean CMT after protocol switch (298.7 ± 66 μm) was not significantly different to the baseline measured before the pandemic period (292.1 ± 89 μm). Mixed effects linear regression demonstrated a non-significant increase of 8.7 μm (SE of 6 μm, *p* = 0.15) after controlling for correlations between subjects (Fig. 1B). The number of eyes with qualitative recordings of IRF, SRF, and SHRM on OCT were not significantly different by McNemar test (Table 1).

**Table 1:**
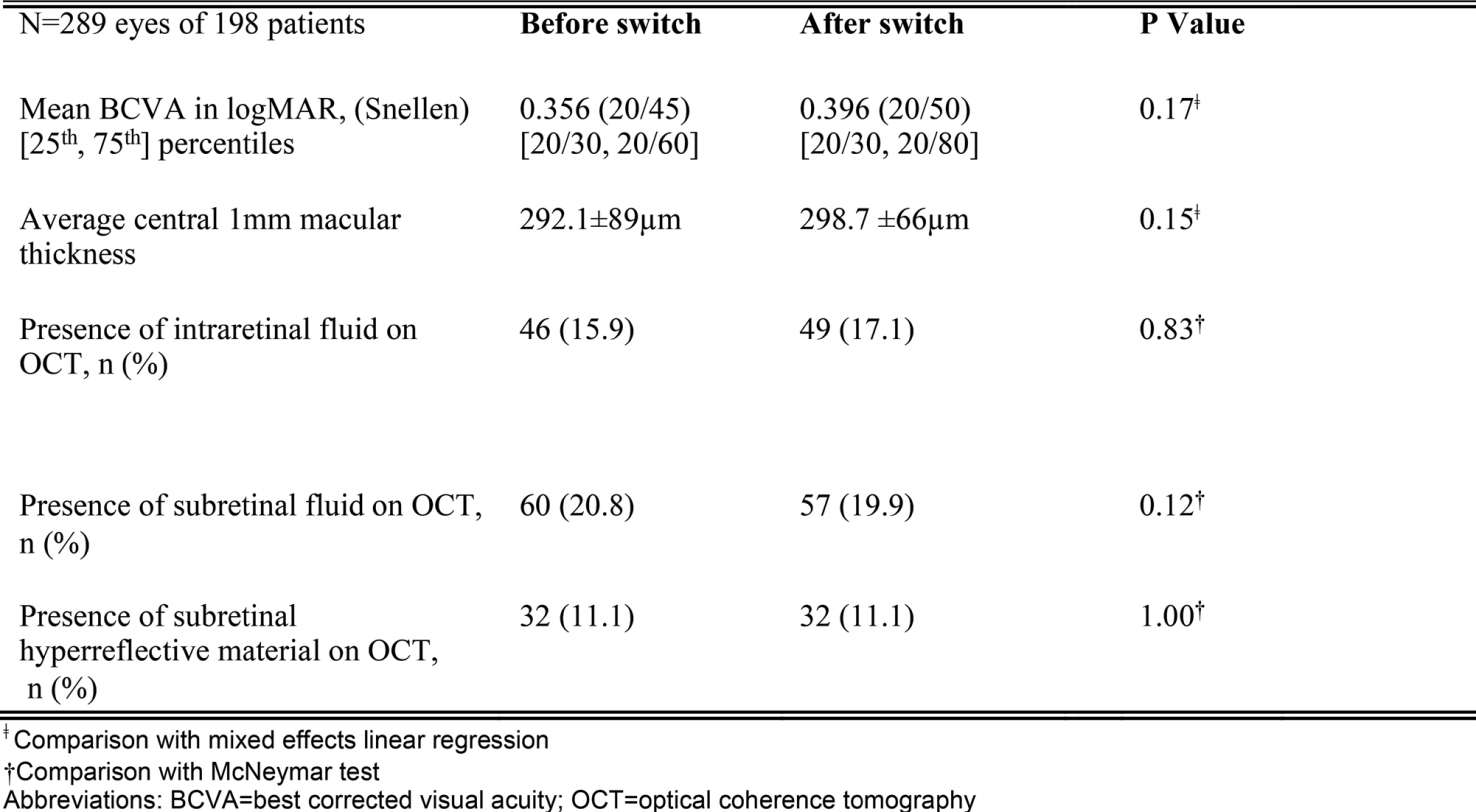
Functional and anatomic outcomes before and after a 3-month change to fixed-interval injections during the early COVID-19 pandemic

### Rates of substantial visual decline

**Fig. 2:**
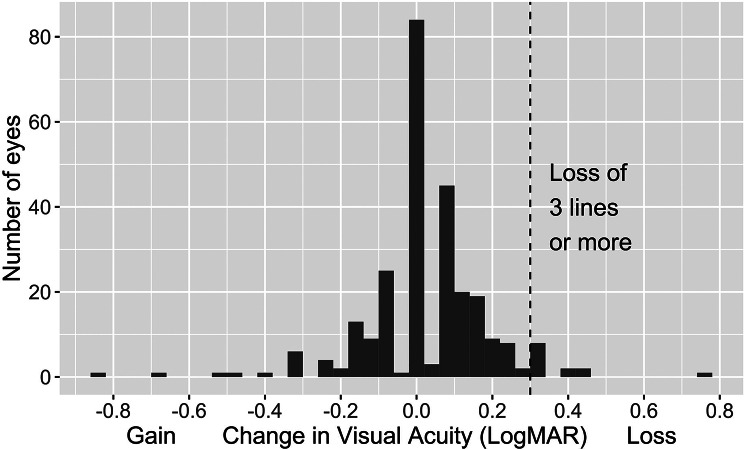
Distribution of VA changes from baseline to follow up after the protocol switch. Difference in logMAR visual acuity (negative = gain, positive = loss) is stratified along the x axis, and the dashed line shows the cutoff representing approximately 3 lines of loss. 9 eyes undergoing maintenance injections (3.3%) lost 3 lines of vision or more, and an additional four eyes had developed new neovascular diasease during the protocol switch period with substantial vision loss. Of the nine eyes with significant vision loss while on maintenance injections, three had missed appointments during the protocol switch period

Nine eyes (3.3%) undergoing maintenance injections in our cohort lost the equivalent of 3 lines or more of visual acuity (LogMAR increased by 0.3) during the follow up period. (Fig. 2)

The average injection interval of these eyes was 7.9 +/- 3.2 weeks. The average interval between the baseline and post-switch follow up visits was 5.7 months, and the incidence of substantial vision loss was 6.0 eyes per 1000 eye-months. Within this subgroup of patients, 4 eyes had decreased VA triggering an OCT but had stable or improving retinal fluid. Given the lack of a control arm in this retrospective study, landmark clinical trials were used as a source of historical data for comparison. In the ANCHOR and MARINA studies, 10% of patients treated with monthly ranibizumab injections lost 15 or more letters of visual acuity over 24 months [20]. This translates to an incidence of substantial vision loss of 4.16 per 1000 eye-months. A chi-square test was performed to compare the incidence in our cohort to the historical value, which showed no significant difference (*p* = 0.43).

### Examples of two clinical cases

**Fig. 3:**
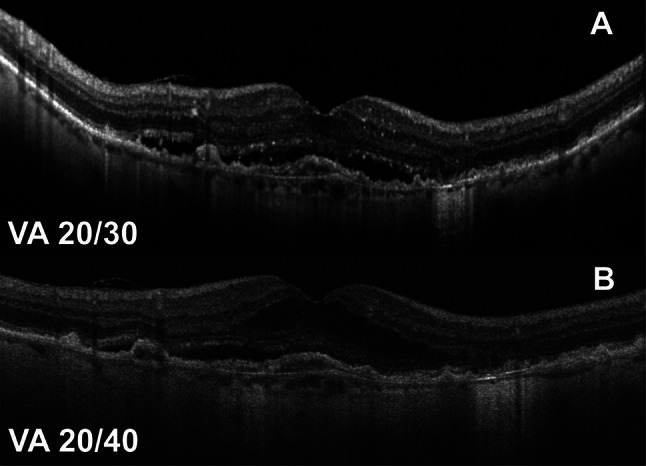
OCT and clinical course of an 85-year-old female with neovascular AMD OU receiving intravitreal bevacizumab. At baseline visit prior to the protocol switch, her VA and Spectralis OCT are shown and she was receiving injections every 6 weeks on treat and extend protocol. (**A**) During the protocol switch, no imaging was obtained in her two follow up visits due to stable visual acuity thus she continued to receive fixed-scheduled intravitreal bevacizumab injections. At her follow up after the protocol switch (**B**), she had mild increase in intraretinal fluid but stability of VA and OCT (Cirrus) still on at the 6-week interval

**Fig. 4:**
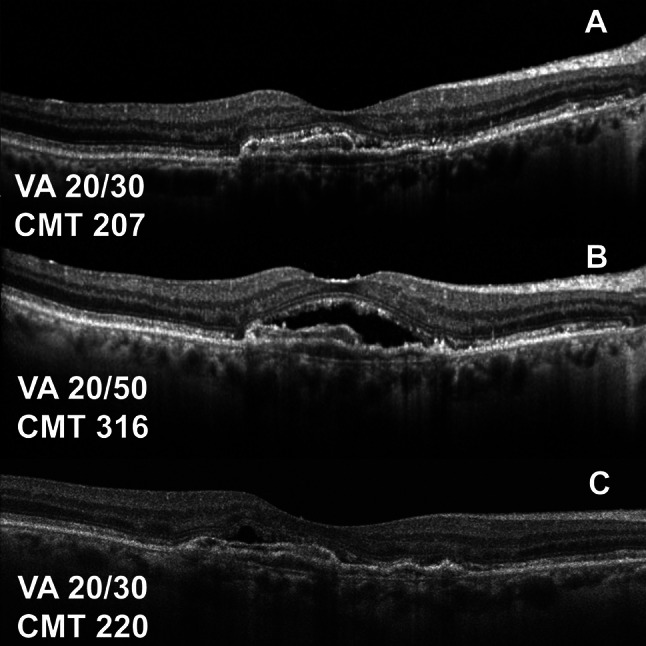
Clinical course during protocol switch with structural OCT of an 87-year-old male with nvAMD on a 6-week interval of intravitreal aflibercept. At baseline visit prior to the protocol switch, his visual acuity (VA) and CMT (Spectralis) are shown (**A**). At first follow up visit during this protocol switch, he had a symptomatic decrease in VA from 20/30 to 20/50. OCT shows an increase in CMT (**B**) to 316 μm thus he was treated with intravitreal aflibercept with a decrease in the follow up interval down to 4 weeks for the remainder of the protocol switch. At the first follow up after protocol switch the patient demonstrated recovery to 20/30 and improved CMT (post-correction on Cirrus) and subretinal fluid (**C**) which was not fully resolved

We highlight the clinical course of two paients suffering from bilateral nvAMD receiving intravitreal anti-VEGF medications during this period. The first case (Fig. 3) highlights a case where the switch to fixed-interval therapy worked well and the second case highlights a patient extended to 6-week interval of intravitreal aflibercept (Fig. 4A) who experienced a decline in VA that necessitated a tightening of his interval to 4 weeks for the remainder of the protocol switch.

### Cancellation or missed visits and death during the early phase of the COVID-19 pandemic

Of the patients who met our inclusion criteria, we found that 19 patients with neovascular AMD were lost to follow up since the start of the pandemic. Of the 189 patients we analyzed, 11 patients tested positive for COVID-19 and 6 of these patients died from COVID-related complications. There were no patients who were diagnosed with COVID-19 within ten days of their last clinic appointment during the protocol switch interval. There were no confirmed or suspected cases of transmission to ophthalmic personnel during this time.

## Discussion

In this study, we evaluated the impact of a planned but forced, abrupt protocol switch from treat-and-extend to fixed-interval anti-VEGF therapy in patients with nvAMD during the start of the COVID-19 pandemic in the US. At that time, the dilemma of trying to preserve vision in patients with nvAMD while adhering to social distancing guidelines and other viral exposure-related limitations was an unprecedented challenge [21]. Under normal circumstances, treat-and-extend and/or individualized regimens have been shown to be beneficial to reduce the treatment burden of intravitreal injections. The Royal College of Ophthalmologists recommended eight-weekly anti-VEGF treatments without clinic reviews or imaging, unless patients reported significant vision loss during injection visits [22]. While fixed-interval treatment is effective for neovascular AMD, it can be burdensome, especially during the pandemic. As a result, some practices shifted to an as-needed approach with phone triage, leading to longer treatment intervals and unavoidable delays for many patients [8, 21, 23].

We instituted a department-wide, fixed-interval injection schedule for our patients and withheld standard imaging such as an OCT unless it was deemed essential to inform treatment and follow up intervals, as outlined above. Reducing testing, which places the patient and photographer in close proximity, was intended to minimize exposure to COVID-19 and potential transmission, especially when personal protective equipment such as masks were of limited availability at the time.

Our goal was to assess short-term outcomes following an abrupt, system-wide shift in treatment strategy. While longer follow-up would provide additional insights, the chosen three-month interval specifically reflects the critical early period during which real-time clinical decisions had to be made under conditions of considerable uncertainty. While this shift was initially driven by pandemic-related considerations, similar fixed-interval strategies may have value in non-pandemic contexts—such as trial maintenance phases, high-volume practices, or settings with access limitations.

In our study, about 5.8% of our patients were documented to be diagnosed with COVID-19 during this time period and nearly half of these patients died from its complications. However, none of our patients contracted COVID-19 within 10 days of their last appointment during the pandemic restriction interval. This indicates that for the duration of the pandemic switch, while hundreds of Iowans were hospitalized and almost 15% of all-cause deaths in the state were attributed to COVID-19, our patients were able to get ongoing care without contracting this infection [24].

Our treatment protocol remained effective in maintaining VA despite the concessions made for safety. There was no significant change in VA or CMT after the three-month period of the protocol switch among eyes undergoing maintenance anti-VEGF treatment who continued to make their appointments. Different studies have shown significant fluctuations of CMT in nvAMD patients in the context of delayed therapy and lockdown implementation, but to our knowledge, this is the first report of CMT stability in AMD patients after implementing a protocol switch during the COVID-19 pandemic [8, 25]. Ultimately, 3.3% of eyes in our cohort had visual acuity loss compared to baseline, which was not significantly increased from the baseline rates of vision loss expected [7, 8, 10, 26, 27]. Furthermore, several of the patients who lost vision had stable OCT imaging with no new fluid and no change in thickness (Fig. 4). Visual acuity measurements were not standardized and may in some cases have been rushed due to the circumstances of the pandemic, further limiting our analysis with a bias toward worsening vision. Even under the most ideal circumstances, there is considerable intersession variability of VA measurements that are most pronounced with advanced AMD [28]. Mild atrophy and/or fibrosis could also be responsible for changes in vision [28].

Our findings are further supported by recent real-world evidence. Kang et al. demonstrated stable visual outcomes over two years despite delays in anti-VEGF injections in nvAMD patients [29]. Additionally, Nanji et al. showed that mild delays in aflibercept treatments resulted in stable visual acuity and anatomical outcomes [30]. These recent studies underscore that short-term fixed-interval regimens with limited imaging do not significantly harm visual outcomes, aligning closely with our observations.

This study has several limitations. The retrospective design and short follow-up period limit long-term generalizability. The conversion algorithm used to harmonize CMT between Cirrus and Spectralis devices was developed for diabetic macular edema and may underestimate CMT in eyes with large PEDs, as the Cirrus RPE fit line lies above Bruch’s membrane. While we excluded scans with obvious segmentation errors, some residual measurement bias is possible. Manual re-segmentation of all Cirrus scans to Bruch’s membrane was not feasible for this dataset; however, targeted re-segmentation in a representative subset could be explored in future studies to quantify the magnitude of this effect. Finally, these findings reflect the anti-VEGF agents available at the time of the COVID-19 protocol change and may not directly apply to newer, longer-acting agents.

Although this protocol change was implemented in response to the COVID-19 pandemic, the findings may have broader relevance for scenarios in which imaging or visit frequency must be reduced—including in clinical trial maintenance phases or practice settings constrained by staffing, access, or patient preference. Such streamlined approaches could improve efficiency in ongoing clinical trials, enhance care delivery for patients facing transportation or access barriers, and support clinical operations during restructuring or staffing shortages.

Overall, we demonstrate that an abrupt protocol change to a fixed-interval treatment with reduced imaging can result in short-term preservation of visual and anatomic outcomes of patients with nvAMD. Given that our safety protocol helped limit viral exposure and maintained visual acuity, we hope our study adds to the body of literature describing outcomes of creative strategies to continue delivering the best care possible should an emergency situation arise again in the future. These findings provide practical insight into how clinics can rapidly adapt treatment paradigms while maintaining patient outcomes.

## Supplementary Information

Below is the link to the electronic supplementary material.

Supplementary Material 1

## Data Availability

The datasets used and/or analysed during the current study are available from the corresponding author on reasonable request.
