# Supplementary material for: Real-world short-term outcomes after switching to fixed-interval anti-VEGF therapy for neovascular AMD during the COVID-19 pandemic
Source: Int J Retina Vitreous. 2025 Oct 8;11:103. doi: 10.1186/s40942-025-00734-w (PMC12505566; doi:10.1186/s40942-025-00734-w)
Supplement: Supplementary file 1 — Supplementary Material 1 [file 40942_2025_734_MOESM1_ESM.docx]

Supplemental Table 1. Characteristics of nvAMD patients and eyes included in analysis

| Number of eyes | 289 |
| --- | --- |
| Number of patients | 198 |
| Eyes receiving   - Bevacizumab - Aflibercept - Ranibizumab - Brolucizumab | 120  119  48  2 |
|  |  |
| Average age (years) | 80.3 |
| Gender, n (%) |  |
| Males | 78 (39%) |
| Females | 120 (61%) |
| Average number of injections during pandemic switch (13-week interval) | 1.89 +/- 0.30 |
| Average number of injections prior to pandemic switch (prior 13-week interval) | 2.12 +/- 0.40 |
